# Anxiety and depression among patients with migraine: A single-center cross-sectional study in Malaysia

**DOI:** 10.1371/journal.pone.0324250

**Published:** 2025-05-27

**Authors:** Sathiapriya Padmanathan, Juen Kiem Tan, Chen Fei Ng, Rathika Rajah, Ching Soong Khoo, Wan Nur Nafisah Wan Yahya, Lai Fong Chan, Michelle Maryanne Tan, Rozita Hod, Hui Jan Tan

**Affiliations:** 1 Department of Medicine, Faculty of Medicine, The National University of Malaysia, Kuala Lumpur, Malaysia; 2 Hospital Canselor Tuanku Muhriz, Cheras, Kuala Lumpur, Malaysia; 3 Department of Psychiatry, Faculty of Medicine, The National University of Malaysia, Kuala Lumpur, Malaysia; 4 Department of Medicine, Hospital Tengku Ampuan Rahimah, Selangor, Malaysia; 5 Department of Public Health Medicine, Faculty of Medicine, The National University of Malaysia, Kuala Lumpur, Malaysia; Universiti Malaya Fakulti Perubatan: University of Malaya Faculty of Medicine, MALAYSIA

## Abstract

**Background:**

Migraine is a chronic neurological problem with a psychological comorbidity. However, anxiety and depression among patients with migraine have not been thoroughly investigated in Southeast Asia. Thus, we aimed to elucidate the prevalence of anxiety and depression in patients with migraine, as well as the associated factors.

**Methods:**

This cross-sectional study was conducted between March 2022 and March 2024 at the National University of Malaysia. The participants’ data were collected prior to completing two questionnaires, including the Patient Health Questionnaire-9 (PHQ-9) and the Generalized Anxiety Disorder-7 (GAD-7), which were used to evaluate depression and anxiety, respectively.

**Results:**

A total of 246 participants who were diagnosed with migraine were recruited for this study. The mean age of the participants was 46.19 years (SD: ± 14.75). Additionally, 77.6% of the participants were female. Moreover, 27.7% of the participants had anxiety alone, 15.9% had depression alone, and 11.8% had both anxiety and depression. A younger age (p = 0.03), earlier age of migraine onset (r(246) -0.178, p < 0.01), background history of asthma (r(246) 0.161, p < 0.05), lower household income range (r(246) -0.179, p < 0.01), increased pain severity (r(246) 0.211, p < 0.01), frequency of attack (r(246) 0.139, p < 0.05), use of NSAIDs (r(246) 0.134, p < 0.05), use of pizotifen (r(246) 0.169, p < 0.01), use of propranolol (r(246) 0.286, p < 0.01), use of sodium valproate (r(246) 0.146, p < 0.05), use of topiramate (r(246) 0.178, p < 0.01), use of more than one medication (r(246) 0.240, p < 0.01), use of cold therapy (r(246) 0.223, p < 0.01) and use of acupuncture (r(246) 0.260, p < 0.01) were associated with anxiety and depression in migraine patients.

**Conclusions:**

Anxiety and depression are commonly observed in patients with migraine in Asia. Routine assessments for anxiety and depression should be performed to ensure holistic management of migraine.

## Introduction

Migraine is a chronic neurological problem characterized by episodic debilitating headache disorders accompanied by nausea, vomiting, photophobia and phonophobia. This disorder can be classified into migraine with aura and migraine without aura. [1] The global migraine prevalence is 14–15% and represents 4.9% of the global population with disability. [2] India, China, the United States and Indonesia have exhibited the highest incidences, accounting for 43.6% of the global population. [3] Migraine is primarily prevalent in women, with a lifetime prevalence of 16.3%, whereas this prevalence is 7% in men. [4] Moreover, a community-based study in Malaysia reported a migraine prevalence of 9%. [5] Another study in Malaysia reported that 61.8% of medical students from a private medical school were potentially diagnosed with migraine using a self-administered questionnaire from the International Headache Society (IHS) criteria [6].

Migraine patients exhibit high burdens of health, [7] economic, [8] and psychosocial disturbances. Anxiety and depression form a complex, intricate interplay with migraine, with these two factors often influencing each other. Research has demonstrated that depression [9] and anxiety [10,11] are more common in individuals with migraine than in healthy individuals. Moreover, psychiatric comorbidities are more common in individuals with chronic migraine than in those with episodic migraine. [12] Additionally, compared with migraine without aura, migraine with aura tends to be associated with psychiatric comorbidities. [11] A bidirectional connection between these conditions has been demonstrated, as individuals with migraine commonly exhibit an association with anxiety and depression; moreover, this comorbidity also increases the risk of migraine [13].

Anxiety disorders are more commonly observed among migraine patients than in the general population, especially regarding those patients with chronic migraine. [4] Migraine, anxiety and depression are closely related due to their similar clinical characteristics, methods of presentation, and episodic natures. Previous studies have reinforced the notion that there is a greater association of anxiety in patients with migraine. [13,14] Stress-induced changes may trigger migraines, thereby leading to hypersensitivity of the limbic system due to neuronal hyperexcitability. [15] A prospective, web-based survey in the United States reported that migraine patients were more likely to develop depression and anxiety, with an odds ratio (OR) of 3.18 (95% confidence interval (CI): 3.0, 3.3) being reported. [16] A case-control study revealed that anxiety and depression were significantly associated with several parameters of migraine, including frequency, severity, disability, impact of the headaches, quality of life and sleep quality [17].

Several pathophysiological factors have been linked with migraine, anxiety and depression; however, the causal mechanism of this association remains unclear. Dopaminergic pathways (particularly those involving the dopamine D2 receptor) and their dysfunction have been attributed to causes of migraine and depression. [18] Migraine and anxiety both involve similar neurotransmitter pathways, such as the 5-hydroxytryptamine, serotonin and glutamatergic pathways. [19] Genetic factors involving the gamma-aminobutyric acid (GABA), dopaminergic, and folate pathways have been implicated as risk factors for migraine with anxiety and depression. Structural brain imaging data from patients with migraine have demonstrated several features similar to those of patients with anxiety and depression. Furthermore, a decrease in gray matter volume has been observed with respect to the anterior cingulate, insula, and prefrontal cortices, which are mainly involved in the nociceptive pathway [20,21].

The comorbidity of anxiety and depression among migraine patients in Southeast Asian countries has not been extensively investigated. A previous study conducted in three Southeast Asian countries (Cambodia, Myanmar and Vietnam) revealed that among chronic diseases, migraines or frequent headaches were positively associated with anxiety and depression. [22] However, this study focused on all chronic diseases, rather than specifically focusing on migraine. Additionally, these Southeast Asian countries differ from Malaysia, which is a tropical country with diverse customs and cultures. There is a possibility that other confounding factors, such as climate, temperature, and food, may aggravate migraine. The large, web-based Migraine in America Symptoms and Treatment (MAST) study was demonstrated to be prone to nonresponse bias, which may have skewed the reported data; moreover, the results cannot be extrapolated to our region, which experiences different types of stigma. [16] Therefore, the demographics and risk factors for psychological comorbidities in migraine patients remain unclear, particularly in this region. Hence, we conducted this study to determine the prevalence and association of anxiety and depression among patients with migraine.

## 2. Materials and methods

### 2.1 Study design and study population

This cross-sectional study was conducted between March 24^th^, 2022, and March 23^rd^, 2024, at Hospital Canselor Tuanku Muhriz, National University of Malaysia. This study was conducted following the guidelines of the Declaration of Helsinki, with written consent being obtained from each participant once they had read the study information sheet and agreed to participate. Study approval was obtained from the National University of Malaysia Ethics and Research Board of the Faculty of Medicine (research code: JEP-2022–188). The study population was recruited via convenience sampling and consisted of patients older than 18-years-old with a diagnosis of migraine for at least a year prior to the study. The diagnosis of migraine was made once the diagnostic criteria stipulated in the International Headache Society (IHS) International Classification of Headache Disorders for migraine were fulfilled. [1] The migraine patients consisted of those who had episodic migraines (fewer than 15 headache days per month) and chronic migraines (at least 15 headache days per month). Patients were excluded from the study if they had secondary headache disorders such as structural brain abnormalities (including brain tumors or strokes), traumatic brain injury, meningoencephalitis, drug/alcohol abuse, malignancy, pregnancy or preexisting psychiatric disorders (including schizophrenia or bipolar mood disorders).

### 2.2 Recruitment and data collection

The selection of the patients was based on specific inclusion criteria. After written informed consent was obtained, data involving patient demographics and clinical migraine history were documented. The demographic variables included age, sex, marital status, education level, income, and clinical data such as medication usage and frequency of attacks. Migraine was classified based on the International Classification of Headache Disorders 3^rd^ edition guidelines for headache disorders. The visual analog scale (VAS) was used to assess the pain perception of the participants during the peak of their migraine attacks. The pain score was determined on a scale of 0–10, with 0 = no pain at all and 10 = most extreme pain. Patients were then required to complete two questionnaires, including the Patient Health Questionnaire-9 (PHQ-9) [23] and the Generalized Anxiety Disorder-7 (GAD-7) [24], in order to evaluate depression and anxiety, respectively. The collected data were subsequently documented in our statistical program for further analysis.

#### 2.2.1 Generalized anxiety disorder-7 (GAD-7).

The GAD-7 questionnaire is a 7-item, self-reported anxiety questionnaire designed to evaluate mental health symptoms. The questionnaire evaluates the degree to which the patient has been affected by feeling nervous, anxious, or on edge; not being able to stop or control worrying; worrying too much about different things; having trouble relaxing; being so restless that it is hard to sit still; becoming easily annoyed or irritable and feeling afraid as if something awful might happen. This scale consists of 7 questions answered on a four-point Likert scale, including scores of 0 (not at all), 1 (several days), 2 (more than half of the days) and 3 (nearly every day). The total score of the seven items on the GAD-7 ranged from 0–21. A total score of 0–4 indicates minimal anxiety, 5–9 indicates mild anxiety, 10–14 indicates moderate anxiety, and 15–21 indicates severe anxiety. This questionnaire is one of the most widely used scales, with pooled sensitivity and specificity values being reported at a cutoff point of 8 [sensitivity: 0.83 (95% CI: 0.71–0.91); specificity: 0.84 (95% CI: 0.70–0.92)]. [25] A systematic review concluded that the GAD-7 demonstrated the best performance characteristics for identifying generalized anxiety disorder (GAD) in comparison to other measures. [26] This assessment is also available in Bahasa Malaysia, with the locally validated Malay GAD-7 cutoff score for anxiety of 8 or greater exhibiting a sensitivity of 76% (95% CI: 61–87%) and specificity of 94% (88–97%). [27] A score of 8 or greater represents the cutoff point for probable cases of generalized anxiety disorder.

#### 2.2.2 Patient Health Questionnaire-9 (PHQ-9).

The Patient Health Questionnaire-9 (PHQ-9) consists of a 9-item depression module that scores each of the 9 DSM-IV criteria as 0 (not at all) to 3 (nearly every day). A PHQ-9 score ≥ 10 exhibits a sensitivity of 88% and a specificity of 88% for major depression. [23] Major depression was diagnosed if 5 or more of the 9 depressive symptom criteria were present at least “more than half of the days” in the past 2 weeks, with 1 of the symptoms involving a depressed mood or anhedonia. The PHQ-9 has been translated into several languages, [28] including the Malay language. The validated Malay version demonstrates a sensitivity of 87% and a specificity of 82% [29].

### 2.3 Statistical analysis

The sample size of the study was calculated based on the formula below, with a confidence level of 95% and a maximum error of 0.05. A prevalence study by Govind et al. involving 206 participants suggested that 20% of patients with migraine alone had anxiety and depression [30].

N = [Z^2^ p (1-p)] / d2

Z= degree of confidence 1.96 (95%)

p: 0.20 d: max error: 0.05

The estimated sample size for this study was 245 patients.

The data were analyzed via SPSS Statistics for Windows version 25 software. Data normality was evaluated using skewness and kurtosis, and nonnormally distributed data are presented as medians ± percentiles for the skewed data and as frequencies (percentages) for the nominal data. The demographic factors and clinical characteristics (categorical variables) were analyzed via the Mann-Whitney test. The relationships between the variables were subsequently analyzed via Spearman correlation analysis.

## 3. Results

We screened a total of 311 patients and recruited 246 participants. Table 1 shows the demographic data of the study population, with a mean age of 46.19 years (SD ± 14.75). The majority of the participants in our cohort were female (191, 77.6%) and had completed tertiary education (208, 84.6%). Common comorbidities observed in the group included hypertension (133 patients, 54.1%), diabetes mellitus (122 patients, 49.6%), dyslipidemia (110 patients, 44.7%), and bronchial asthma (26 patients, 10.6%), as well as ischemic heart disease, chronic kidney disease, previous stroke or transient ischemic attack, and epilepsy. The mean age of onset of migraine was 46.19 years (SD ± 14.75, 95% CI: 44.34–48.04), with the median duration of migraine attacks recorded as 24 hours (24, 48). The median frequency of attacks was three per month (2, 5), and the median number of days of absenteeism per year was 3 (0.0, 5.0). Additionally, the median pain score on the VAS was 8 (7, 8), and the median number of prescribed antimigraine (pharmacological and nonpharmacological) therapies was 2 (2, 3). Table 2 provides a list of the various migraine treatments that were used by the study participants. Notably, analgesia use was more prevalent among the study cohort. The most commonly used antimigraine drugs in the study cohort included sumatriptan (47, 19.1%), ergotamine (32, 13%), propranolol (61, 24.8%), amitriptyline (57, 23.2%), duloxetine (32, 13%), pizotifen (25, 10.2%) and erenumab (31, 12.6%). Additionally, cold compression was the predominant nonpharmacological treatment method utilized in the cohort (120, 48.8%).

**Table 1:** Sociodemographic characteristics among study participants with migraine.

Risk Factors	No. of participants, N (%)	Depression		Anxiety		Depression and Anxiety	
		Yes, N (%)	No, N (%)	Yes, N (%)	No, N (%)	Yes, N (%)	No, N (%)
Gender							
Male	55 (22.4)	8 (3.3)	47 (19.1)	13 (5.3)	42 (17.1)	5 (2.0)	50 (20.3)
Female	191 (77.6)	31 (12.6)	160 (65.0)	55 (22.4)	136 (55.3)	24 (9.8)	167 (67.9)
Race							
Malay	175 (71.1)	29 (11.8)	146 (59.3)	56 (22.8)	119 (48.4)	23 (9.3)	152 (61.8)
Chinese	47 (19.1)	6 (2.4)	41 (16.7)	4 (1.6)	43 (17.5)	3 (1.2)	44 (17.9)
Indian	20 (8.1)	3 (1.2)	17 (6.9)	7 (2.8)	13 (5.3)	2 (0.8)	8 (7.3)
Others	4 (1.6)	1 (0.4)	3 (1.2)	1 (0.4)	3 (1.2)	1 (0.4)	3(1.2)
Age (years)							
18–44	130 (52.8)	21 (8.5)	109 (44.3)	41 (16.7)	89 (36.2)	18 (7.3)	112 (45.5)
45–64	80 (32.5)	15 (6.1)	65 (26.4)	21 (8.5)	59 (24.0)	10 (4.1)	70 (28.5)
>65	36 (14.6)	3 (1.2)	33 (13.4)	6 (2.4)	30 (12.2)	1 (0.4)	35 (14.2)
Education Level							
None	4 (1.6)	0 (0.0)	4 (1.6)	0 (0.0)	4 (1.6)	0 (0.0)	4 (1.6)
Primary	2 (0.8)	1 (0.4)	1 (0.4)	0 (0.0)	2 (0.8)	0 (0.0)	2 (0.8)
Secondary	32 (13.0)	8 (3.3)	24 (9.8)	13 (5.3)	19 (7.7)	5 (2.0)	27 (11.0)
Tertiary	208 (84.6)	30 (12.2)	178 (72.4)	55 (22.4)	153 (62.2)	24 (9.8)	184 (74.8)
Income							
<RM2,500	17 (6.9)	4 (1.6)	13 (5.3)	8 (3.3)	9 (3.7)	3 (1.2)	14 (5.7)
RM2,501–RM10,000	155 (63.0)	33 (13.4)	122 (49.6)	51 (20.7)	104 (42.3)	24 (9.8)	131 (53.3)
>RM10,000	74 (30.1)	2 (0.8)	72 (29.3)	9 (3.7)	65 (26.4)	2 (0.8)	72 (29.3)
Comorbidities:							
Hypertension							
No	113 (45.9)	17 (6.9)	96 (39.0)	36 (14.6)	77 (31.3)	13 (5.3)	100 (40.7)
Yes	133 (54.1)	22 (8.9)	111 (45.1)	32 (13.0)	101 (41.1)	16 (6.5)	117 (47.6)
Diabetes Mellitus							
No	124 (50.4)	15 (6.1)	24 (9.8)	32 (13.0)	92 (37.4)	12 (4.9)	112 (45.5)
Yes	122 (49.6)	109 (44.3)	98 (39.8)	36 (14.6)	86 (35.0)	17 (6.9)	105 (42.7)
IHD							
No	240 (97.6)	38 (15.4)	202 (82.1)	68 (27.6)	172 (69.9)	29 (11.8)	211 (85.8)
Yes	6 (2.4)	1 (0.4)	5 (2.0)	0 (0.0)	6 (2.4)	0 (0.0)	6 (2.4)
Dyslipidemia							
No	136 (55.3)	14 (5.7)	122 (49.6)	35 (14.2)	101 (41.1)	13 (5.3)	123 (50.0)
Yes	110 (44.7)	25 (10.2)	85 (34.6)	33 (13.4)	77 (31.3)	16 (6.5)	94 (38.2)
Bronchial Asthma							
No	220 (89.4)	29 (11.8)	191 (77.6)	53 (21.5)	167 (67.9)	22 (8.9)	198 (80.5)
Yes	26 (10.6)	10 (4.1)	16 (6.5)	15 (6.1)	11 (4.5)	7 (2.8)	19 (7.7)
CKD							
No	244 (99.2)	39 (15.9)	205 (83.3)	68 (27.6)	176 (71.5)	29 (11.8)	215 (87.4)
Yes	2 (0.8)	0 (0.0)	2 (0.8)	0 (0.0)	2 (0.8)	0 (0.0)	2 (0.8)
Previous stroke/TIA							
No	241 (98.0)	39 (15.9)	202 (82.1)	68 (27.6)	173 (70.3)	29 (11.8)	212 (86.2)
Yes	5 (2.0)	0 (0.0)	5 (2.0)	0 (0.0)	5 (2.0)	0 (0.0)	5 (2.0)
Epilepsy							
No	244 (99.2)	39 (15.9)	205 (83.3)	68 (27.6)	176 (71.5)	29 (11.8)	215 (87.4)
Yes	2 (0.8)	0 (0.0)	2 (0.8)	0 (0.0)	2 (0.8)	0 (0.0)	2 (0.8)
Number of medications:							
1	8 (3.3)	0 (0.0)	8 (3.3)	0 (0.0)	8 (3.3)	0 (0.0)	8 (3.3)
2–3	146 (59.3)	11 (4.5)	135 (54.9)	27 (11.0)	119 (48.4)	9 (3.7)	137 (55.7)
≥ 4	92 (37.4)	28 (11.4)	64 (26.0)	41 (16.7)	51 (20.7)	20 (8.1)	72 (29.3)
			**Mean, SD (95% CI)**				
Age			46.19 ± 14.75 (44.34–48.04)				
Number of antimigraine medications			3.02 ± 1.13 (2.88–3.17)				
			**Median (Percentile 25th, 75th)**				
Age of onset			30.0 (20.0, 40.0)				
Duration of migraine attack			24.0 (24.0, 48.0)				
Frequency of migraine attack			3.0 (2.0, 5.0)				
Days of absenteeism			3.0 (0.0, 5.0)				
Pain scale			8.0 (7.0, 8.0)				

CKD - Chronic Kidney Disease.

IHD - Ischemic Heart Disease.

TIA - Transient Ischemic Attack.

SD - Standard Deviation.

CI - Confidence Interval.

RM - Ringgit Malaysia.

**Table 2:** Antimigraine therapy usage among the study cohort.

Treatment Option		No. of participants, N (%)	Depression		Anxiety		Depression and Anxiety	
			No, N (%)	Yes, N (%)	No, N (%)	Yes, N (%)	No, N (%)	Yes, N (%)
**Pharmacological:**								
Paracetamol	No	29 (11.8)	23 (9.3)	6 (2.4)	26 (10.6)	3 (1.2)	26 (10.6)	3 (1.2)
	Yes	217 (88.2)	155 (63)	62 (25.2)	181 (73.6)	36 (14.6)	191 (77.6)	26 (10.6)
NSAIDs	No	169 (68.7)	129 (52.4)	40 (16.3)	152 (61.8)	17 (6.9)	154 (62.6)	15 (6.1)
	Yes	77 (31.3)	49 (19.9)	28 (11.4)	55 (22.4)	22 (8.9)	63 (25.6)	14 (5.7)
Sumatriptan	No	199 (80.9)	143 (58.1)	56 (22.8)	168 (68.3)	31 (12.6)	176 (71.5)	23 (9.3)
	Yes	47 (19.1)	35 (14.2)	12 (4.9)	39 (15.9)	8 (3.3)	41 (16.7)	6 (2.4)
Ergotamine	No	214 (87)	159 (64.6)	55 (22.4)	183 (74.4)	31 (12.6)	190 (77.2)	24 (9.8)
	Yes	32 (13)	19 (7.7)	13 (5.3)	24 (9.8)	8 (3.3)	27 (11)	5 (2)
Tramadol	No	216 (87.8)	159 (64.6)	57 (23.2)	181 (73.6)	35 (14.2)	191 (77.6)	25 (10.2)
	Yes	30 (12.2)	19 (7.7)	11 (4.5)	26 (10.6)	4 (1.6)	10 (4.1)	10 (4.1)
Propranolol	No	185 (75.2)	148 (60.2)	37 (15)	167 (67.9)	18 (7.3)	173 (70.3)	12 (4.9)
	Yes	61 (24.8)	30 (12.2)	31 (12.6)	40 (16.3)	21 (8.5)	44 (17.9)	17 (6.9)
Amitriptyline	No	189 (76.8)	135 (54.9)	54 (22)	162 (65.9)	27 (11)	169 (68.7)	20 (8.1)
	Yes	57 (23.2)	43 (17.5)	14 (5.7)	45 (18.3)	12 (4.9)	48 (19.5)	9 (3.7)
Duloxetine	No	214 (87)	159 (64.6)	55 (22.4)	184 (74.8)	30 (12.2)	190 (77.2)	24 (9.8)
	Yes	32 (13)	19 (7.7)	13 (5.3)	23 (9.3_)	9 (3.7)	27 (11)	5 (2)
Pizotifen	No	221 (89.8)	164 (66.7)	57 (23.2)	192 (78)	29 (11.8)	199 (80.9)	22 (8.9)
	Yes	25 (10.2)	14 (5.7)	11 (4.5)	15 (6.1)	10 (4.1)	18 (7.3)	7 (2.8)
Topiramate	No	227 (92.3)	170 (69.1)	57 (23.2)	199 (80.9)	28 (11.4)	204 (82.9)	23 (9.3)
	Yes	19 (7.7)	8 (3.3)	11 (4.5)	8 (3.3)	11 (4.5)	13 (5.3)	6 (2.4)
Sodium Valproate	No	238 (96.7)	174 (70.7)	64 (26)	204 (82.9)	34 (13.8)	212 (86.2)	26 (10.6)
	Yes	8 (3.3)	4 (1.6)	4 (1.6)	3 (1.2)	5 (2.0)	5 (2)	3 (1.2)
Flunarizine	No	232 (94.3)	167 (67.9)	65 (26.4)	196 (79.7)	36 (14.6)	205 (83.3)	27 (11)
	Yes	14 (5.7)	11 (4.5)	3 (1.2)	11 (4.5)	3 (1.2)	12 (4.9)	2 (0.8)
ACEi/ARB	No	244 (99.2)	177 (72)	67 (27.2)	205 (83.3)	39 (15.9)	215 (87.4)	29 (11.8)
	Yes	2 (0.8)	1 (0.4)	1 (0.4)	2 (0.8)	0 (0)	2 (0.8)	0 (0)
Erenumab	No	215 (87.4)	154 (62.6)	61 (24.8)	179 (72.8)	36 (14.6)	188 (76.4)	27 (11)
	Yes	31 (12.6)	24 (9.8)	7 (2.8)	28 (11.4)	3 (1.2)	29 (11.8)	2 (0.8)
**Non-Pharmacological:**								
Acupuncture	No	226 (91.9)	168 (68.3)	58 (23.6)	198 (80.5)	28 (11.4)	205 (83.3)	21 (8.5)
	Yes	20 (8.1)	10 (4.1)	10 (4.1)	9 (3.7)	11 (4.5)	12 (4.9)	8 (3.3)
Herbal	No	221 (89.8)	158 (64.2)	63 (25.6)	188 (76.4)	33 (13.4)	196 (79.7)	25 (10.2)
	Yes	25 (10.2)	20 (8.1)	5 (2)	19 (7.7)	6 (2.4)	21 (8.5)	4 (1.6)
Cold Compression	No	126 (51.2)	109 (44.3)	17 (6.9)	118 (48)	8 (3.3)	120 (48.8)	6 (2.4)
	Yes	120 (48.8)	69 (28)	51 (20.7)	89 (36.2)	31 (12.6)	97 (39.4)	23 (9.3)
Botox	No	246 (100)	178 (72.4)	68 (27.6)	207 (84.1)	39 (15.9)	217 (88.2)	28 (11.8)
Transmagnetic Stimulation	No	246 (100)	178 (72.4)	68 (27.6)	207 (84.1)	39 (15.9)	217 (88.2)	29 (11.8)

NSAIDs - Nonsteroidal Anti-inflammatory Drugs.

ACEi - Angiotensin Converting Enzyme Inhibitor.

ARB - Angiotensin Receptor Blocker.

The associations between sociodemographic factors and anxiety and depression within the study cohort are detailed in Table 3. Age of migraine onset, household income, and pain scale score were significantly associated with anxiety and depression among the study cohort (p < 0.05). Bronchial asthma was the only comorbidity that was significantly associated with anxiety and depression in our cohort (p = 0.003). However, there was no significant association observed between anxiety or depression and sex, race, education level, history of previous stroke or history of transient ischemic attack among our study participants.

**Table 3:** Associations of sociodemographic, clinical factors and various treatment strategies with anxiety and depression.

Variables	Anxiety & Depression	Mean Rank	U	Z	p Value
Gender	negative	124.34	2964.000	-0.703	0.482
	positive	117.21			
Race	negative	124.66	2895.500	-0.877	0.381
	positive	114.84			
Education Level	negative	123.73	3097.500	-0.217	0.828
	positive	121.81			
Income Range	negative	127.44	2291.000	-2.797	**0.005**
	positive	94.00			
Hypertension	negative	123.32	3107.000	-0.127	0.899
	positive	124.86			
Diabetes Mellitus	negative	122.02	2824.500	-1.033	0.302
	positive	134.60			
Ischemic Heart Disease	negative	123.90	3059.500	-0.905	0.366
	positive	120.50			
Hyperlipidemia	negative	121.78	2773.500	-1.203	0.229
	positive	136.36			
Asthma	negative	121.27	2662.500	-2.525	**0.012**
	positive	140.19			
Chronic Kidney Disease	negative	123.63	3117.500	-0.518	0.604
	positive	122.50			
Previous Stroke or TIA	negative	123.83	3074.000	-0.824	0.410
	positive	121.00			
Epilepsy	negative	123.63	3117.500	-0.518	0.604
	positive	122.50			
Psychiatric Disease	negative	123.97	3045.000	-0.979	0.327
	positive	120.00			
Age of Onset	negative	128.12	2144.000	-2.794	**0.005**
	positive	88.93			
Duration of Attack	negative	124.29	2974.500	-0.509	0.611
	positive	117.57			
Frequency of Attack	negative	119.94	2374.500	-2.175	**0.030**
	positive	150.12			
Days of Absenteeism/year	negative	121.91	2801.000	-0.993	0.321
	positive	135.41			
Pain Scale (VAS)	negative	118.21	1998.500	-3.303	**0.001**
	positive	163.09			
Paracetamol	negative	123.26	3095.000	-0.256	0.798
	positive	125.28			
NSAIDs	negative	120.71	2541.000	-2.095	**0.036**
	positive	144.38			
Sumatriptan	negative	123.24	3090.000	-0.231	0.818
	positive	125.45			
Ergotamine	negative	122.80	2995.500	-0.720	0.471
	positive	128.71			
Tramadol	negative	123.24	3089.500	-0.279	0.780
	positive	125.47			
Acupuncture	negative	120.30	2452.500	-4.074	**0.000**
	positive	147.43			
Herbal	negative	122.90	3017.000	-0.688	0.492
	positive	127.97			
Cold Compression	negative	118.48	2057.500	-3.495	**0.000**
	positive	161.05			
Propranolol	negative	117.94	1940.000	-4.482	**0.000**
	positive	165.10			
Amitriptyline	negative	122.21	2866.000	-1.066	0.286
	positive	133.17			
Duloxetine	negative	122.80	2995.500	-0.720	0.471
	positive	128.71			
Pizotifen	negative	121.20	2648.000	-2.647	**0.008**
	positive	140.69			
Topiramate	negative	121.37	2684.000	-2.779	**0.005**
	positive	139.45			
Sodium Valproate	negative	122.33	2893.500	-2.288	**0.022**
	positive	132.22			
Flunarizine	negative	123.30	3103.500	-0.298	0.766
	positive	124.98			
ACEi/ARB	negative	123.63	3117.500	-0.518	0.604
	positive	122.50			
Erenumab	negative	124.44	2943.000	-0.984	0.325
	positive	116.48			
Number of medications	negative	118.15	1984.500	-3.757	**0.000**
	positive	163.57			
**Independent t Test**					
			**t**	**df**	**p**
Age			1.839	244	**0.034**

p value significant at <0.05.

U – Mann-Whitney test.

Z – Z value.

t - t statistics.

NSAIDs - Nonsteroidal Anti-inflammatory Drugs.

ACEi - Angiotensin Converting Enzyme Inhibitor.

ARB - Angiotensin Receptor Blocker.

As demonstrated in Table 3, an independent sample t test was used to analyze the associations between age and the occurrences of anxiety and depression. Age (M = 46.19, SD: ± 14.75) was significantly associated with anxiety and depression (t(244) = 1.893, p = 0.03).

Spearman’s correlation analysis was performed to examine the relationships regarding sex, race, education level, income range, hypertension, diabetes mellitus, ischemic heart disease, dyslipidemia, asthma, chronic kidney disease, previous stroke, previous transient ischemic attack, epilepsy, psychiatric disease, age of onset, attack duration, frequency of attack days of absenteeism per year and pain scale scores with anxiety and depression. As shown in Table 4, the results of the Spearman correlation indicated a weak but statistically significant negative relationship regarding income range (r(246): -0.179, p < 0.01) and age of onset (r(246): -0.178, p < 0.01) with anxiety and depression. Additionally, a very weak positive relationship was observed regarding asthma (r(246): 0.161, p < 0.05), the frequency of migraine attack (r(246): 0.139, p < 0.05), and pain severity (r(246): 0.211, p < 0.01) with the presence of anxiety and depression.

**Table 4:** Relationship between sociodemographic, clinical features and migraine treatment with anxiety and depression.

	1	2	3	4	5	6	7	8	9	10	11	12
1. Anxiety & Depression												
2. Income range	**-0.179** ^**^											
3. Asthma	**0.161** ^*^	-0.213^**^										
4. Age of Onset	**-0.178** ^**^	0.12	0.11									
5. Days of Absenteeism/year	0.06	0.09	-0.02	-0.265^**^								
6. Paracetamol	0.02	-0.03	0.126^*^	-0.07	0.11							
7. NSAIDs	**0.134** ^*^	0.03	-0.06	-0.02	0.04	-0.161^*^						
8. Ergotamine	0.05	-0.143^*^	0.299^**^	0.04	-0.04	-0.01	-0.03					
9. Tramadol	0.02	-0.03	-0.05	0.00	0.11	-0.02	-0.04	-0.07				
10. Acupuncture	**0.260** ^**^	-0.08	0.236^**^	-0.07	0.07	0.06	0.12	0.06	-0.02			
11. Propranolol	**0.286** ^**^	-0.325^**^	0.323^**^	-0.07	-0.07	0.01	0.10	0.06	-0.07	0.277^**^		
12. Topiramate	**0.178** ^**^	-0.223^**^	0.198^**^	0.00	0.04	0.01	0.166^**^	0.02	-0.01	0.248^**^	0.222^**^	
13. Number of medications	**0.240** ^**^	-0.308^**^	0.227^**^	0.00	0.07	0.09	0.288^**^	0.09	0.131^*^	0.317^**^	0.382^**^	0.306^**^

*Correlation is significant at the 0.05 level (2-tailed).

**Correlation is significant at the 0.01 level (2-tailed).

NSAIDs - Nonsteroidal Anti-inflammatory drugs.

Spearman’s correlation was also performed for various migraine therapies. The number of utilized medications was weakly positively correlated with anxiety and depression. In addition, a weak positive relationship was observed regarding NSAIDs (r(246): 0.134, p < 0.05), propranolol (r(246): 0.286, p < 0.01), pizotifen (r(246): 0.169, p < 0.01), topiramate (r(246): 0.178, p < 0.01) and sodium valproate (r(246): 0.146, p < 0.05) with anxiety and depression. Nonpharmacological therapies such as acupuncture (r(246): 0.260, p < 0.01) and cold compression (r(246): 0.223, p < 0.01) also demonstrated a weakly positive relationship with anxiety and depression, which were statistically significant.

## 4. Discussion

Our study of 246 participants revealed that 27.7% of the participants had anxiety alone, 15.9% had depression alone, and 11.8% had both anxiety and depression. The identified associated factors included the age of the participants, age of onset, lower household income range, background bronchial asthma, pain severity, frequency of attack, and the uses of medications such as NSAIDs, pizotifen, propranolol, sodium valproate, topiramate, multiple medications, cold therapy and acupuncture. These results will be further discussed in the following sections.

### 4.1 Prevalence

In the Global Burden of Diseases, Injuries and Risk Factors (GBD) studies, 1.04 billion individuals were estimated to have migraine. [31] Migraine has been well documented to be associated with psychiatric comorbidities. Patients with migraine exhibit 2–10 times higher rates of depression and anxiety compared with the general population. [32] Similarly, our study reported that the prevalence rates of anxiety and depression in migraine patients were 27.7% and 15.9%, respectively. The reported prevalence of anxiety in migraine patients ranges from 19% to 44.5%. [33,34] Additionally, the incidence of depression in migraine patients ranges from 8.6% to 47.9% according to the results of a previous meta-analysis. [14] Moreover, a cross-sectional epidemiological study in Singapore revealed that migraine headache was significantly associated with psychiatric conditions such as major depressive disorder (prevalence ratio (PR): 1.80; 95% CI: 1.25–2.58) and generalized anxiety disorder (PR: 2.04; 95% CI: 1.12–3.69) [35].

The prevalence of both anxiety and migraine in our cohort was 11.8%. This finding is comparable to that of a study by Oh et al. in South Korea, which reported a prevalence of 11.6%. [34] Anxiety and depression were also observed to be significantly independently associated with an increased risk of migraine and migraine-related burdens. [17] A Taiwanese study reported that chronic migraine patients exhibited increased risks of depression (RR = 1.88; p < 0.001) and anxiety disorders (RR = 1.48; p < 0.001). [36] The variation in the reported prevalence rates is largely attributed to the study design, population, and screening methods utilized by the studies.

### 4.2 Sociodemographic factors

The mean age of our cohort was 46.19 years, whereas 77.6% of the participants were female. These findings are consistent with other studies in which the prevalence of migraine peaks between the ages of 20–40 years and is the main reason for disability in young women. [37] Our study revealed that age (p = 0.005) and age at migraine onset (p = 0.034) were associated with anxiety and depression. Moreover, we observed a weakly negative but statistically significant (r (246): -0.178, p < 0.01) correlation between age of onset and anxiety and depression, thereby suggesting that patients with earlier ages of onset and younger patients with migraine were likely to be anxious and depressed. These results are consistent with previous studies reporting greater risks of anxiety and depression in children and adolescents with migraines compared with healthy controls. [38] Similar findings of increased prevalence of psychiatric comorbidities (particularly depression and anxiety) in adolescents have also been reported in other studies. [39,40] The contributing factors for these findings may include lifestyle changes, the use of electronic devices, sleep deprivation and psychological stress. [41,42]

Total household income was another factor associated with anxiety and depression in migraine patients (p = 0.005). Our study demonstrated a weak negative correlation with income range (r(246): -0.179, p < 0.01). Data extracted from the American Migraine Prevalence and Prevention Study involving 25,000 participants revealed increases in the prevalences of migraine and attack frequency in those with lower household incomes. [43] In addition, income dissatisfaction is more frequently observed in those individuals with lower incomes. Furthermore, income dissatisfaction has been linked to increases in migraine incidence and the frequency of attack, which are both linked to anxiety and depression [44].

A premorbid history of bronchial asthma was also associated with anxiety and depression in migraine patients (p = 0.003), wherein a weak but positive correlation was noted (r(246): 0.161, p < 0.05). Asthma and migraine have long been theorized to share a bidirectional relationship. [45] A study on asthma and migraine conducted by Kim et al. reported an adjusted hazard ratio of 1.47 for migraine (95% CI: 1.41–1.53, p < 0.001) in the asthma group compared with the control group. Additionally, a meta-analysis of fifteen studies involving 1,188,780 participants further supported the link between asthma and an increased incidence of migraine (hazard ratio: 1.47; 95% CI: 1.41 ~ 1.52). [46] The exact mechanism underlying this link has not been determined, although it may be related to parasympathetic hyperreactivity. Importantly, patients with asthma alone are more prone to anxiety and depressive symptoms [47].

### 4.3 Clinical factors

Our study demonstrated an association regarding pain severity based on the VAS score (p = 0.001) and the frequency of migraine attacks (p = 0.030) with anxiety and depression in migraine patients. Correlation analysis revealed a weakly positive relationship of pain severity (r(246): 0.211, p < 0.01) and frequency of attack (r(246): 0.139, p < 0.05) with anxiety and depression. This study concurs with a previous study reporting that pain severity is a predictor of anxiety in migraine patients. [48] Likewise, anxiety could be a trigger of headache activity, as it has been reported to be significantly associated with headache. [49] However, another study revealed that migraine intensity was an important anxiety factor, with a moderate frequency of headaches and sex both representing risk factors for anxiety and depression. [50] The variability demonstrated in pain perception in migraine patients has also been supported in a systemic review, which revealed an increase in pain perception intensity during the ictal phase compared with the interictal phase. [51] This scenario could be potentiated in the presence of anxiety symptoms, which may lead to alterations in emotional status and the response to pain. [52] Moreover, these observations may explain the increase in pain severity that is observed in migraine patients, particularly in those with anxiety and depression.

A study conducted in Spain reported a positive linear correlation between the number of headache days per month and the risk of anxiety. [53] Duan et al. reported that anxiety and depression were significantly independently associated with migraine frequency, severity, disability, headache impact, quality of life and sleep quality in migraine patients. [17] Furthermore, the Chronic Migraine Epidemiology and Outcomes (CaMEO) study revealed that depression and anxiety are associated with a greater rate of headache-related disability [54].

The use of medications such as NSAIDs was observed to be associated with anxiety and depression in our cohort (p = 0.036). NSAIDs represented the most commonly prescribed medication (taken by 31.3% of the participants) and demonstrated a weakly positive correlation with anxiety and depression (r(246): 0.134, p < 0.05). The analgesic property of this medication is supported by indirect evidence of prostaglandin involvement. [55] NSAIDs function by inhibiting cyclooxygenase, which is the enzyme that catalyzes the synthesis of prostaglandins. An increase in prostaglandins has been linked with an increased incidence of depression. [56] Additionally, the frequent use of NSAIDs can also lead to medication-overuse headaches, thus leading to an increased frequency of headaches. Conversely, a study reported that patients who responded to NSAIDs exhibited significantly lower anxiety and depression scores. [57] A previous cross-sectional study revealed that patients with medication-overuse headaches demonstrated higher scores of anxiety and depression than those who did not use excessive medications for acute relief [58].

Commonly used migraine prophylactics, such as pizotifen (p = 0.008) and propranolol (p < 0.001), were associated with anxiety and depression in migraine patients, with a weakly positive relationship being observed (pizotifen, r(246): 0.169; propranolol, r(246): 0.286; p < 0.01). Propranolol is a lipophilic drug and can more readily cross the blood-brain barrier compared with other beta-blockers. [59] Although medications such as pizotifen have been linked with neurobehavioral and depressive symptoms, the relationship between beta-blockers and an increased prevalence of depression is more likely biased than causal, according to its indication for use in mood disorders [60,61].

Antiseizure medications that are administered as migraine prophylaxis, such as sodium valproate (p = 0.022) and topiramate (p = 0.005), were found to be associated with anxiety and depression in our cohort, with both medications demonstrating a weakly positive correlation (sodium valproate, r(246): 0.146, p < 0.05; topiramate, r(246): 0.178, p < 0.01). Both medications are commonly used as adjuncts in the management of mood disorders and depression. However, they have also been linked with increases in suicidal ideation and negative symptoms, which is possibly due to their effects on GABA neurotransmission or interactions with monoaminergic systems [62–64].

Interestingly, many patients in our cohort also resorted to nonpharmacological therapeutic methods in addition to medications. Cold compression (p < 0.001) and acupuncture (p < 0.001) were observed to be significantly associated with anxiety and depression in migraine patients. The proposed mechanism of cold therapy involves the activation of the sympathetic nervous system, increased blood levels of beta-endorphin and noradrenaline, and increased synaptic release of noradrenaline in the brain. [65] A case-control study in which cold therapy was applied to patients with migraine reported improvements in the visual analog scale score twenty-five minutes after initiating treatment of the migraine attack. [66] Additionally, the exact mechanism by which acupuncture assists in the alleviation of pain and prophylaxis of migraine is still unclear, although systemic reviews support its effects [67].

However, our results demonstrated that cold compress therapy was an associated factor for anxiety and depression in migraine patients. This association may be explained by the fact that unpredictable attacks of severe migraine headaches may lead to anxiety and depression. Thus, patients may resort to other alternative therapies (such as cold compresses) as adjunctive therapy.

### Limitations

We acknowledge several limitations in this study. For example, there was no separate analysis of the diagnosis of migraine and other medical conditions. As this was a single-center study, we cannot extrapolate the findings to the overall community. In addition, we were unable to identify individual risk factors due to the study design. In the future, we plan to conduct a multicenter, case-control study to strengthen research in this field. Furthermore, the use of self-rated screening tools such as the PHQ-9 and GAD-7 could lead to recall bias and the possibility of misinterpretation of results due to cultural and sociodemographic differences.

## Conclusion

We observed an 11.8% prevalence of anxiety and depression in patients with migraine. This study also revealed that the factors associated with anxiety and depression in migraine patients in this region are comparable to those factors reported in the Western literature. Younger patients, earlier age of migraine onset, background history of asthma, lower household income range, increased pain severity, increased frequency of attack, and the use of medications such as NSAIDs, pizotifen, propranolol, sodium valproate, and topiramate (along with the use of more than one type of medication) were associated with anxiety and depression in migraine patients. Interestingly, cold therapy and acupuncture demonstrated a weakly positive relationship with anxiety and depression; however, this relationship is more likely biased than causal. This study highlights the need to consider the impact on a specific patient’s psychological health to ensure holistic management; moreover, by utilizing this information, the clinical benefits can also be considered.

## Supporting information

S1 Table Sociodemographic characteristics of the study participants with migraine. (DOCX)

S2 Table Antimigraine therapy usage among the study cohort. (DOCX)

S3 Table Associations of sociodemographic factors, clinical factors and various treatment strategies with anxiety and depression. (DOCX)

S4 Table Relationship between sociodemographic, clinical features and migraine treatment with anxiety and depression. (DOCX)

## List of abbreviations:

OR: odds ratio
CI: confidence interval
GABA: gamma-aminobutyric acid
RM: Ringgit Malaysia
VAS: visual analog scale
PHQ-9: Patient Health Questionnaire-9
GAD-7: Generalized Anxiety Disorder-7
DSM-IV: Diagnostic and Statistical Manual of Mental Disorders, Fourth edition
NSAIDs: Nonsteroidal anti-inflammatory drugs
