# Supplementary material for: Anxiety and depression among patients with migraine: A single-center cross-sectional study in Malaysia
Source: PLoS One. 2025 May 27;20(5):e0324250. doi: 10.1371/journal.pone.0324250 (PMC12111257; doi:10.1371/journal.pone.0324250)
Supplement: S1 Table — (DOCX) [file pone.0324250.s001.docx]

| **Table 1: Sociodemographic characteristics among study participants with migraine** | | | | | | | |
| --- | --- | --- | --- | --- | --- | --- | --- |
| Risk Factors | No. of participants, N (%) | Depression | | Anxiety | | Depression and Anxiety | |
|  |  | Yes,  N (%) | No,  N (%) | Yes,  N (%) | No,  N (%) | Yes,  N (%) | No,  N (%) |
| Gender |  |  |  |  |  |  |  |
| Male | 55 (22.4) | 8 (3.3) | 47 (19.1) | 13 (5.3) | 42 (17.1) | 5 (2.0) | 50 (20.3) |
| Female | 191 (77.6) | 31 (12.6) | 160 (65.0) | 55 (22.4) | 136 (55.3) | 24 (9.8) | 167 (67.9) |
|  |  |  |  |  |  |  |  |
| Race |  |  |  |  |  |  |  |
| Malay | 175 (71.1) | 29 (11.8) | 146 (59.3) | 56 (22.8) | 119 (48.4) | 23 (9.3) | 152 (61.8) |
| Chinese | 47 (19.1) | 6 (2.4) | 41 (16.7) | 4 (1.6) | 43 (17.5) | 3 (1.2) | 44 (17.9) |
| Indian | 20 (8.1) | 3 (1.2) | 17 (6.9) | 7 (2.8) | 13 (5.3) | 2 (0.8) | 8 (7.3) |
| Others | 4 (1.6) | 1 (0.4) | 3 (1.2) | 1 (0.4) | 3 (1.2) | 1 (0.4) | 3(1.2) |
|  |  |  |  |  |  |  |  |
| Age (years) |  |  |  |  |  |  |  |
| 18–44 | 130 (52.8) | 21 (8.5) | 109 (44.3) | 41 (16.7) | 89 (36.2) | 18 (7.3) | 112 (45.5) |
| 45–64 | 80 (32.5) | 15 (6.1) | 65 (26.4) | 21 (8.5) | 59 (24.0) | 10 (4.1) | 70 (28.5) |
| >65 | 36 (14.6) | 3 (1.2) | 33 (13.4) | 6 (2.4) | 30 (12.2) | 1 (0.4) | 35 (14.2) |
|  |  |  |  |  |  |  |  |
| Education Level |  |  |  |  |  |  |  |
| None | 4 (1.6) | 0 (0.0) | 4 (1.6) | 0 (0.0) | 4 (1.6) | 0 (0.0) | 4 (1.6) |
| Primary | 2 (0.8) | 1 (0.4) | 1 (0.4) | 0 (0.0) | 2 (0.8) | 0 (0.0) | 2 (0.8) |
| Secondary | 32 (13.0) | 8 (3.3) | 24 (9.8) | 13 (5.3) | 19 (7.7) | 5 (2.0) | 27 (11.0) |
| Tertiary | 208 (84.6) | 30 (12.2) | 178 (72.4) | 55 (22.4) | 153 (62.2) | 24 (9.8) | 184 (74.8) |
|  |  |  |  |  |  |  |  |
| Income |  |  |  |  |  |  |  |
| <RM2,500 | 17 (6.9) | 4 (1.6) | 13 (5.3) | 8 (3.3) | 9 (3.7) | 3 (1.2) | 14 (5.7) |
| RM2,501–RM10,000 | 155 (63.0) | 33 (13.4) | 122 (49.6) | 51 (20.7) | 104 (42.3) | 24 (9.8) | 131 (53.3) |
| >RM10,000 | 74 (30.1) | 2 (0.8) | 72 (29.3) | 9 (3.7) | 65 (26.4) | 2 (0.8) | 72 (29.3) |
|  |  |  |  |  |  |  |  |
| Comorbidities: |  |  |  |  |  |  |  |
| Hypertension |  |  |  |  |  |  |  |
| No | 113 (45.9) | 17 (6.9) | 96 (39.0) | 36 (14.6) | 77 (31.3) | 13 (5.3) | 100 (40.7) |
| Yes | 133 (54.1) | 22 (8.9) | 111 (45.1) | 32 (13.0) | 101 (41.1) | 16 (6.5) | 117 (47.6) |
|  |  |  |  |  |  |  |  |
| Diabetes Mellitus |  |  |  |  |  |  |  |
| No | 124 (50.4) | 15 (6.1) | 24 (9.8) | 32 (13.0) | 92 (37.4) | 12 (4.9) | 112 (45.5) |
| Yes | 122 (49.6) | 109 (44.3) | 98 (39.8) | 36 (14.6) | 86 (35.0) | 17 (6.9) | 105 (42.7) |
|  |  |  |  |  |  |  |  |
| IHD |  |  |  |  |  |  |  |
| No | 240 (97.6) | 38 (15.4) | 202 (82.1) | 68 (27.6) | 172 (69.9) | 29 (11.8) | 211 (85.8) |
| Yes | 6 (2.4) | 1 (0.4) | 5 (2.0) | 0 (0.0) | 6 (2.4) | 0 (0.0) | 6 (2.4) |
|  |  |  |  |  |  |  |  |
| Dyslipidemia |  |  |  |  |  |  |  |
| No | 136 (55.3) | 14 (5.7) | 122 (49.6) | 35 (14.2) | 101 (41.1) | 13 (5.3) | 123 (50.0) |
| Yes | 110 (44.7) | 25 (10.2) | 85 (34.6) | 33 (13.4) | 77 (31.3) | 16 (6.5) | 94 (38.2) |
|  |  |  |  |  |  |  |  |
| Bronchial Asthma |  |  |  |  |  |  |  |
| No | 220 (89.4) | 29 (11.8) | 191 (77.6) | 53 (21.5) | 167 (67.9) | 22 (8.9) | 198 (80.5) |
| Yes | 26 (10.6) | 10 (4.1) | 16 (6.5) | 15 (6.1) | 11 (4.5) | 7 (2.8) | 19 (7.7) |
|  |  |  |  |  |  |  |  |
| CKD |  |  |  |  |  |  |  |
| No | 244 (99.2) | 39 (15.9) | 205 (83.3) | 68 (27.6) | 176 (71.5) | 29 (11.8) | 215 (87.4) |
| Yes | 2 (0.8) | 0 (0.0) | 2 (0.8) | 0 (0.0) | 2 (0.8) | 0 (0.0) | 2 (0.8) |
|  |  |  |  |  |  |  |  |
| Previous stroke/TIA |  |  |  |  |  |  |  |
| No | 241 (98.0) | 39 (15.9) | 202 (82.1) | 68 (27.6) | 173 (70.3) | 29 (11.8) | 212 (86.2) |
| Yes | 5 (2.0) | 0 (0.0) | 5 (2.0) | 0 (0.0) | 5 (2.0) | 0 (0.0) | 5 (2.0) |
|  |  |  |  |  |  |  |  |
| Epilepsy |  |  |  |  |  |  |  |
| No | 244 (99.2) | 39 (15.9) | 205 (83.3) | 68 (27.6) | 176 (71.5) | 29 (11.8) | 215 (87.4) |
| Yes | 2 (0.8) | 0 (0.0) | 2 (0.8) | 0 (0.0) | 2 (0.8) | 0 (0.0) | 2 (0.8) |
|  |  |  |  |  |  |  |  |
| Number of medications: |  |  |  |  |  |  |  |
| 1 | 8 (3.3) | 0 (0.0) | 8 (3.3) | 0 (0.0) | 8 (3.3) | 0 (0.0) | 8 (3.3) |
| 2 to 3 | 146 (59.3) | 11 (4.5) | 135 (54.9) | 27 (11.0) | 119 (48.4) | 9 (3.7) | 137 (55.7) |
| More than 4 | 92 (37.4) | 28 (11.4) | 64 (26.0) | 41 (16.7) | 51 (20.7) | 20 (8.1) | 72 (29.3) |
|  |  |  |  |  |  |  |  |
|  |  |  |  |  |  |  |  |
|  |  |  | **Mean, SD (95% CI)** | | | | |
| Age | | | 46.19 ± 14.75 (44.34–48.04) | | | | |
| Number of antimigraine medications | | | 3.02 ± 1.13 (2.88–3.17) | | | | |
|  |  |  |  |  |  |  |  |
|  |  |  | **Median (Percentile 25th, 75th)** | | | | |
| Age of onset | | | 30.0 (20.0, 40.0) | | | | |
| Duration of migraine attack | | | 24.0 (24.0, 48.0) | | | | |
| Frequency of migraine attack | | | 3.0 (2.0, 5.0) | | | | |
| Days of absenteeism | | | 3.0 (0.0, 5.0) | | | | |
| Pain scale | | | 8.0 (7.0, 8.0) | | | | |
|  | | | | | | | |
| CKD - Chronic Kidney Disease IHD - Ischemic Heart Disease TIA - Transient Ischemic Attack SD - Standard Deviation CI - Confidence Interval | | | | | | | |
