# Supplementary material for: Anxiety and depression among patients with migraine: A single-center cross-sectional study in Malaysia
Source: PLoS One. 2025 May 27;20(5):e0324250. doi: 10.1371/journal.pone.0324250 (PMC12111257; doi:10.1371/journal.pone.0324250)
Supplement: S2 Table — (DOCX) [file pone.0324250.s002.docx]

| **Table 2: Antimigraine therapy usage among the study cohort** | | | | | | | | |
| --- | --- | --- | --- | --- | --- | --- | --- | --- |
| Treatment Option |  | No. of participants,  N (%) | Depression | | Anxiety | | Depression and Anxiety | |
|  |  |  | No,  N (%) | Yes,  N (%) | No,  N (%) | Yes,  N (%) | No,  N (%) | Yes,  N (%) |
| **Pharmacological:** | | | | | | | | |
| Paracetamol | No | 29 (11.8) | 23 (9.3) | 6 (2.4) | 26 (10.6) | 3 (1.2) | 26 (10.6) | 3 (1.2) |
|  | Yes | 217 (88.2) | 155 (63) | 62 (25.2) | 181 (73.6) | 36 (14.6) | 191 (77.6) | 26 (10.6) |
|  |  |  |  |  |  |  |  |  |
| NSAIDs | No | 169 (68.7) | 129 (52.4) | 40 (16.3) | 152 (61.8) | 17 (6.9) | 154 (62.6) | 15 (6.1) |
|  | Yes | 77 (31.3) | 49 (19.9) | 28 (11.4) | 55 (22.4) | 22 (8.9) | 63 (25.6) | 14 (5.7) |
|  |  |  |  |  |  |  |  |  |
| Sumatriptan | No | 199 (80.9) | 143 (58.1) | 56 (22.8) | 168 (68.3) | 31 (12.6) | 176 (71.5) | 23 (9.3) |
|  | Yes | 47 (19.1) | 35 (14.2) | 12 (4.9) | 39 (15.9) | 8 (3.3) | 41 (16.7) | 6 (2.4) |
|  |  |  |  |  |  |  |  |  |
| Ergotamine | No | 214 (87) | 159 (64.6) | 55 (22.4) | 183 (74.4) | 31 (12.6) | 190 (77.2) | 24 (9.8) |
|  | Yes | 32 (13) | 19 (7.7) | 13 (5.3) | 24 (9.8) | 8 (3.3) | 27 (11) | 5 (2) |
|  |  |  |  |  |  |  |  |  |
| Tramadol | No | 216 (87.8) | 159 (64.6) | 57 (23.2) | 181 (73.6) | 35 (14.2) | 191 (77.6) | 25 (10.2) |
|  | Yes | 30 (12.2) | 19 (7.7) | 11 (4.5) | 26 (10.6) | 4 (1.6) | 10 (4.1) | 10 (4.1) |
|  |  |  |  |  |  |  |  |  |
| Propranolol | No | 185 (75.2) | 148 (60.2) | 37 (15) | 167 (67.9) | 18 (7.3) | 173 (70.3) | 12 (4.9) |
|  | Yes | 61 (24.8) | 30 (12.2) | 31 (12.6) | 40 (16.3) | 21 (8.5) | 44 (17.9) | 17 (6.9) |
|  |  |  |  |  |  |  |  |  |
| Amitriptyline | No | 189 (76.8) | 135 (54.9) | 54 (22) | 162 (65.9) | 27 (11) | 169 (68.7) | 20 (8.1) |
|  | Yes | 57 (23.2) | 43 (17.5) | 14 (5.7) | 45 (18.3) | 12 (4.9) | 48 (19.5) | 9 (3.7) |
|  |  |  |  |  |  |  |  |  |
| Duloxetine | No | 214 (87) | 159 (64.6) | 55 (22.4) | 184 (74.8) | 30 (12.2) | 190 (77.2) | 24 (9.8) |
|  | Yes | 32 (13) | 19 (7.7) | 13 (5.3) | 23 (9.3_) | 9 (3.7) | 27 (11) | 5 (2) |
|  |  |  |  |  |  |  |  |  |
| Pizotifen | No | 221 (89.8) | 164 (66.7) | 57 (23.2) | 192 (78) | 29 (11.8) | 199 (80.9) | 22 (8.9) |
|  | Yes | 25 (10.2) | 14 (5.7) | 11 (4.5) | 15 (6.1) | 10 (4.1) | 18 (7.3) | 7 (2.8) |
|  |  |  |  |  |  |  |  |  |
| Topiramate | No | 227 (92.3) | 170 (69.1) | 57 (23.2) | 199 (80.9) | 28 (11.4) | 204 (82.9) | 23 (9.3) |
|  | Yes | 19 (7.7) | 8 (3.3) | 11 (4.5) | 8 (3.3) | 11 (4.5) | 13 (5.3) | 6 (2.4) |
|  |  |  |  |  |  |  |  |  |
| Sodium Valproate | No | 238 (96.7) | 174 (70.7) | 64 (26) | 204 (82.9) | 34 (13.8) | 212 (86.2) | 26 (10.6) |
|  | Yes | 8 (3.3) | 4 (1.6) | 4 (1.6) | 3 (1.2) | 5 (2.0) | 5 (2) | 3 (1.2) |
|  |  |  |  |  |  |  |  |  |
| Flunarizine | No | 232 (94.3) | 167 (67.9) | 65 (26.4) | 196 (79.7) | 36 (14.6) | 205 (83.3) | 27 (11) |
|  | Yes | 14 (5.7) | 11 (4.5) | 3 (1.2) | 11 (4.5) | 3 (1.2) | 12 (4.9) | 2 (0.8) |
|  |  |  |  |  |  |  |  |  |
| ACEi/ARB | No | 244 (99.2) | 177 (72) | 67 (27.2) | 205 (83.3) | 39 (15.9) | 215 (87.4) | 29 (11.8) |
|  | Yes | 2 (0.8) | 1 (0.4) | 1 (0.4) | 2 (0.8) | 0 (0) | 2 (0.8) | 0 (0) |
|  |  |  |  |  |  |  |  |  |
| Erenumab | No | 215 (87.4) | 154 (62.6) | 61 (24.8) | 179 (72.8) | 36 (14.6) | 188 (76.4) | 27 (11) |
|  | Yes | 31 (12.6) | 24 (9.8) | 7 (2.8) | 28 (11.4) | 3 (1.2) | 29 (11.8) | 2 (0.8) |
|  |  |  |  |  |  |  |  |  |
| **Non-Pharmacological:** | | | | | | | | |
| Acupuncture | No | 226 (91.9) | 168 (68.3) | 58 (23.6) | 198 (80.5) | 28 (11.4) | 205 (83.3) | 21 (8.5) |
|  | Yes | 20 (8.1) | 10 (4.1) | 10 (4.1) | 9 (3.7) | 11 (4.5) | 12 (4.9) | 8 (3.3) |
|  |  |  |  |  |  |  |  |  |
| Herbal | No | 221 (89.8) | 158 (64.2) | 63 (25.6) | 188 (76.4) | 33 (13.4) | 196 (79.7) | 25 (10.2) |
|  | Yes | 25 (10.2) | 20 (8.1) | 5 (2) | 19 (7.7) | 6 (2.4) | 21 (8.5) | 4 (1.6) |
|  |  |  |  |  |  |  |  |  |
| Cold Compression | No | 126 (51.2) | 109 (44.3) | 17 (6.9) | 118 (48) | 8 (3.3) | 120 (48.8) | 6 (2.4) |
|  | Yes | 120 (48.8) | 69 (28) | 51 (20.7) | 89 (36.2) | 31 (12.6) | 97 (39.4) | 23 (9.3) |
|  |  |  |  |  |  |  |  |  |
| Botox | No | 246 (100) | 178 (72.4) | 68 (27.6) | 207 (84.1) | 39 (15.9) | 217 (88.2) | 28 (11.8) |
|  |  |  |  |  |  |  |  |  |
| Transmagnetic Stimulation | No | 246 (100) | 178 (72.4) | 68 (27.6) | 207 (84.1) | 39 (15.9) | 217 (88.2) | 29 (11.8) |
|  |  |  |  |  |  |  |  |  |
| NSAIDs - Nonsteroidal Anti-inflammatory Drugs ACEi - Angiotensin Converting Enzyme Inhibitor ARB - Angiotensin Receptor Blocker | | | | | | | | |
