# Supplementary material for: Anxiety and depression among patients with migraine: A single-center cross-sectional study in Malaysia
Source: PLoS One. 2025 May 27;20(5):e0324250. doi: 10.1371/journal.pone.0324250 (PMC12111257; doi:10.1371/journal.pone.0324250)
Supplement: S3 Table — (DOCX) [file pone.0324250.s003.docx]

| **Table 3:** **Associations of sociodemographic, clinical factors and various treatment strategies with anxiety and depression** | | | | | | | |
| --- | --- | --- | --- | --- | --- | --- | --- |
| Variables | | Anxiety & Depression | | Mean Rank | U | Z | p Value |
| Gender | | negative | | 124.34 | 2964.000 | -0.703 | 0.482 |
|  | | positive | | 117.21 |  |  |  |
| Race | | negative | | 124.66 | 2895.500 | -0.877 | 0.381 |
|  | | positive | | 114.84 |  |  |  |
| Education Level | | negative | | 123.73 | 3097.500 | -0.217 | 0.828 |
|  | | positive | | 121.81 |  |  |  |
| Income Range | | negative | | 127.44 | 2291.000 | -2.797 | **0.005** |
|  | | positive | | 94.00 |  |  |  |
| Hypertension | | negative | | 123.32 | 3107.000 | -0.127 | 0.899 |
|  | | positive | | 124.86 |  |  |  |
| Diabetes Mellitus | | negative | | 122.02 | 2824.500 | -1.033 | 0.302 |
|  | | positive | | 134.60 |  |  |  |
| Ischemic Heart Disease | | negative | | 123.90 | 3059.500 | -0.905 | 0.366 |
|  | | positive | | 120.50 |  |  |  |
| Hyperlipidemia | | negative | | 121.78 | 2773.500 | -1.203 | 0.229 |
|  | | positive | | 136.36 |  |  |  |
| Asthma | | negative | | 121.27 | 2662.500 | -2.525 | **0.012** |
|  | | positive | | 140.19 |  |  |  |
| Chronic Kidney Disease | | negative | | 123.63 | 3117.500 | -0.518 | 0.604 |
|  | | positive | | 122.50 |  |  |  |
| Previous Stroke or TIA | | negative | | 123.83 | 3074.000 | -0.824 | 0.410 |
|  | | positive | | 121.00 |  |  |  |
| Epilepsy | | negative | | 123.63 | 3117.500 | -0.518 | 0.604 |
|  | | positive | | 122.50 |  |  |  |
| Psychiatric Disease | | negative | | 123.97 | 3045.000 | -0.979 | 0.327 |
|  | | positive | | 120.00 |  |  |  |
| Age of Onset | | negative | | 128.12 | 2144.000 | -2.794 | **0.005** |
|  | | positive | | 88.93 |  |  |  |
| Duration of Attack | | negative | | 124.29 | 2974.500 | -0.509 | 0.611 |
|  | | positive | | 117.57 |  |  |  |
| Frequency of Attack | | negative | | 119.94 | 2374.500 | -2.175 | **0.030** |
|  | | positive | | 150.12 |  |  |  |
| Days of Absenteeism/year | | negative | | 121.91 | 2801.000 | -0.993 | 0.321 |
|  | | positive | | 135.41 |  |  |  |
| Pain Scale (VAS) | | negative | | 118.21 | 1998.500 | -3.303 | **0.001** |
|  | | positive | | 163.09 |  |  |  |
| Paracetamol | | negative | | 123.26 | 3095.000 | -0.256 | 0.798 |
|  | | positive | | 125.28 |  |  |  |
| NSAIDs | | negative | | 120.71 | 2541.000 | -2.095 | **0.036** |
|  | | positive | | 144.38 |  |  |  |
| Sumatriptan | | negative | | 123.24 | 3090.000 | -0.231 | 0.818 |
|  | | positive | | 125.45 |  |  |  |
| Ergotamine | | negative | | 122.80 | 2995.500 | -0.720 | 0.471 |
|  | | positive | | 128.71 |  |  |  |
| Tramadol | | negative | | 123.24 | 3089.500 | -0.279 | 0.780 |
|  | | positive | | 125.47 |  |  |  |
| Acupuncture | | negative | | 120.30 | 2452.500 | -4.074 | **0.000** |
|  | | positive | | 147.43 |  |  |  |
| Herbal | | negative | | 122.90 | 3017.000 | -0.688 | 0.492 |
|  | | positive | | 127.97 |  |  |  |
| Cold Compression | | negative | | 118.48 | 2057.500 | -3.495 | **0.000** |
|  | | positive | | 161.05 |  |  |  |
| Propranolol | | negative | | 117.94 | 1940.000 | -4.482 | **0.000** |
|  | | positive | | 165.10 |  |  |  |
| Amitriptyline | | negative | | 122.21 | 2866.000 | -1.066 | 0.286 |
|  | | positive | | 133.17 |  |  |  |
| Duloxetine | | negative | | 122.80 | 2995.500 | -0.720 | 0.471 |
|  | | positive | | 128.71 |  |  |  |
| Pizotifen | | negative | | 121.20 | 2648.000 | -2.647 | **0.008** |
|  | | positive | | 140.69 |  |  |  |
| Topiramate | | negative | | 121.37 | 2684.000 | -2.779 | **0.005** |
|  | | positive | | 139.45 |  |  |  |
| Sodium Valproate | | negative | | 122.33 | 2893.500 | -2.288 | **0.022** |
|  | | positive | | 132.22 |  |  |  |
| Flunarizine | | negative | | 123.30 | 3103.500 | -0.298 | 0.766 |
|  | | positive | | 124.98 |  |  |  |
| ACEi/ARB | | negative | | 123.63 | 3117.500 | -0.518 | 0.604 |
|  | | positive | | 122.50 |  |  |  |
| Erenumab | | negative | | 124.44 | 2943.000 | -0.984 | 0.325 |
|  | | positive | | 116.48 |  |  |  |
| Number of medications | | negative | | 118.15 | 1984.500 | -3.757 | **0.000** |
|  | | positive | | 163.57 |  |  |  |
| **Independent t Test** | | | | | | | |
|  |  | |  | | **t** | **df** | **p** |
| Age |  | |  | | 1.839 | 244 | **0.034** |
|  |  | |  | |  |  |  |
| p value significant at <0.05 U – Mann-Whitney test Z – Z value t - t statistics NSAIDs - Nonsteroidal Anti-inflammatory Drugs ACEi - Angiotensin Converting Enzyme Inhibitor ARB - Angiotensin Receptor Blocker | | | | | | | |
