# Supplementary material for: Anxiety and depression among patients with migraine: A single-center cross-sectional study in Malaysia
Source: PLoS One. 2025 May 27;20(5):e0324250. doi: 10.1371/journal.pone.0324250 (PMC12111257; doi:10.1371/journal.pone.0324250)
Supplement: S4 Table — (DOCX) [file pone.0324250.s004.docx]

| **Table 4:** **Relationship between sociodemographic, clinical features and migraine treatment with anxiety and depression** | | | | | | | | | | | | |
| --- | --- | --- | --- | --- | --- | --- | --- | --- | --- | --- | --- | --- |
|  | 1.00 | 2.00 | 3.00 | 4.00 | 5.00 | 6.00 | 7.00 | 8.00 | 9.00 | 10.00 | 11.00 | 12.00 |
| 1. Anxiety & Depression |  |  |  |  |  |  |  |  |  |  |  |  |
| 2. Income range | **-0.179^**^** |  |  |  |  |  |  |  |  |  |  |  |
| 3. Asthma | **0.161^*^** | -0.213^**^ |  |  |  |  |  |  |  |  |  |  |
| 4. Age of Onset | **-0.178^**^** | 0.12 | 0.11 |  |  |  |  |  |  |  |  |  |
| 5. Days of Absenteeism/year | 0.06 | 0.09 | -0.02 | -0.265^**^ |  |  |  |  |  |  |  |  |
| 6. Paracetamol | 0.02 | -0.03 | 0.126^*^ | -0.07 | 0.11 |  |  |  |  |  |  |  |
| 7. NSAIDs | **0.134^*^** | 0.03 | -0.06 | -0.02 | 0.04 | -0.161^*^ |  |  |  |  |  |  |
| 8. Ergotamine | 0.05 | -0.143^*^ | 0.299^**^ | 0.04 | -0.04 | -0.01 | -0.03 |  |  |  |  |  |
| 9. Tramadol | 0.02 | -0.03 | -0.05 | 0.00 | 0.11 | -0.02 | -0.04 | -0.07 |  |  |  |  |
| 10. Acupuncture | **0.260^**^** | -0.08 | 0.236^**^ | -0.07 | 0.07 | 0.06 | 0.12 | 0.06 | -0.02 |  |  |  |
| 11. Propranolol | **0.286^**^** | -0.325^**^ | 0.323^**^ | -0.07 | -0.07 | 0.01 | 0.10 | 0.06 | -0.07 | 0.277^**^ |  |  |
| 12.Topiramate | **0.178^**^** | -0.223^**^ | 0.198^**^ | 0.00 | 0.04 | 0.01 | 0.166^**^ | 0.02 | -0.01 | 0.248^**^ | 0.222^**^ |  |
| 13. Number of medications | **0.240^**^** | -0.308^**^ | 0.227^**^ | 0.00 | 0.07 | 0.09 | 0.288^**^ | 0.09 | 0.131^*^ | 0.317^**^ | 0.382^**^ | 0.306^**^ |
| *. Correlation is significant at the 0.05 level (2-tailed). **. Correlation is significant at the 0.01 level (2-tailed). NSAIDs - Nonsteroidal Anti-inflammatory Drugs | | | | | | | | | | | | |
